# Regulation of protein thermal stability and its potential application in the development of thermo-attenuated vaccines

**DOI:** 10.1016/j.engmic.2024.100162

**Published:** 2024-06-25

**Authors:** Maofeng Wang, Cancan Wu, Nan Liu, Xiaoqiong Jiang, Hongjie Dong, Shubao Zhao, Chaonan Li, Sujuan Xu, Lichuan Gu

**Affiliations:** a State Key Laboratory of Microbial Technology, Shandong University, Qingdao 266237, China; b Research Center of Translational Medicine, Central Hospital Affiliated to Shandong First Medical University, Jinan 250013, China; c Department of Orthopedics, Shandong Provincial Hospital Affiliated to Shandong First Medical University, Jinan 250021, China; d Shandong Institute of Parasitic Diseases, Shandong First Medical University & Shandong Academy of Medical Sciences, Jining 272033, China

**Keywords:** Protein melting temperature, Enzyme activity, SARS-CoV-2 N protein, 3CL^pro^, Temperature sensitive attenuated vaccines

## Abstract

The coronavirus disease 2019 (COVID-19) pandemic has highlighted the importance of developing novel vaccines. An ideal vaccine should trigger an intense immune reaction without causing significant side effects. In this study we found that substitution of tryptophan located in the cores of severe acute respiratory syndrome coronavirus 2 (SARS-CoV-2) protein structures with certain smaller amino acids resulted in variants with melting temperatures of 33–37 °C. An enzyme activity assay indicated that the proteolytic activity of the main proteinase (3CL^pro^) decreased sharply when the environmental temperature exceeded the melting temperature, implying that other protein variants may lose most of their functions under the same conditions. This finding suggests that a virus variant containing engineered proteins with melting temperatures of 33–37 °C may only be functional in the upper respiratory tract where the temperature is about 33 °C, but will be unable to invade internal organs, which maintain temperatures above 37 °C, thus making it possible to construct temperature-sensitive attenuated vaccines.

## Highlights

• This research has successfully devised a technique to lower the denaturation temperature of the crucial SARS-CoV-2 protein on purpose by modifying specific amino acids within its structural core.
• The correlation between protein denaturation temperature and function was established.
• Targeted reduction of protein thermal stability has the potential to be used to develop attenuated vaccines that are sensitive to temperature.

## Graphical abstract

**Figure:**
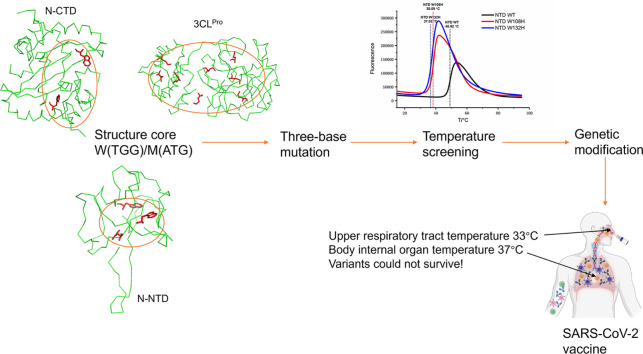

## 1 Introduction

The coronavirus disease 2019 (COVID-19) pandemic caused by severe acute respiratory syndrome coronavirus 2 (SARS-CoV-2) has posed a severe threat to human health and the global economy owing to its high incidence and mortality rate [1,2]. Although the virus has evolved to attenuate illness severity [3], it still poses a significant health risk [4]. Development of effective vaccines is crucial for protecting human health and emphasizing the importance of innovation in vaccine development [5,6]. To date, multiple vaccine modalities have been used to contain the spread of the virus [7]. The mRNA vaccines utilize viral genetic information, intramuscularly injected to induce an immune response to expressed viral antigens [8]. Protein subunit vaccines utilize key portions of the virus, such as the spike protein, to activate the immune system [9,10]. Traditional inactivated and vector-borne vaccines have also been widely used [11,12]. Although these vaccines have achieved success, they have revealed certain drawbacks and limitations. Nucleic acid vaccines can cause adverse reactions [13,14]. Inactivated vaccines require stringent production conditions and carry the risk of incomplete virus inactivation [12]. Protein subunit vaccines may elicit weaker immunogenicity [15]. These vaccines may not always provide optimal immune protection, necessitating ongoing research and innovation to enhance vaccine efficacy and safety.

Traditional attenuated vaccines have been successfully used to prevent many infectious diseases because of their high immunogenicity and low cost. However, these vaccines were not utilized in the fight against the COVID-19 pandemic, probably because traditional attenuated vaccines are developed through multiple passages and natural mutation screening, which are time-consuming, leading to reversion mutations and widespread infection [16].

Temperature-sensitive mutant vaccines emerged more than half a century ago. These vaccines rely on viral mutants which are unable to replicate or cause infection at high temperatures of the vital internal organs [17]. This approach offers the potential to enhance vaccine safety profiles while inducing a robust immune response in the upper respiratory tract, which is the primary site of viral entry and replication. Many live temperature-sensitive mutant vaccines have been selected by repeatedly subculturing the virus at cool (26–29 °C) temperatures either in chicken embryos or in tissue culture [[18], [19], [20]]. Examples of vaccines developed using this approach include the Sabin polio vaccine [21], the rubella vaccine (Meruvax II) [22], and the FluMist intranasal vaccine [19]. However, this approach remains dependent on random mutations and cannot exclude the possibility of back mutations.

We aimed to obtain a temperature-sensitive attenuated vaccine through targeted modification rather than natural passage, to save time and reduce the probability of reversion mutations. Previous studies have shown that substitutions of specific amino acids can alter protein stability and melting temperatures [23]. We found that substitution of tryptophan located in the core of folded SARS-CoV-2 proteins with certain smaller amino acids resulted in variants with melting temperatures of 33–37 °C. Among the 20 amino acids that constitute proteins, only tryptophan and methionine are encoded by non-degenerate codons, thus having the lowest probability of reversion mutations. By strategically replacing tryptophan and methionine with smaller amino acids, protein variants with reduced melting temperatures within the range corresponding to the upper respiratory tract temperature, and corresponding vaccines can be easily created.

## 2 Materials and methods

### 2.1 Plasmid construction for N protein and 3CL^pro^

Genes encoding the N protein and 3CL^pro^ with codon optimization were commercially synthesized. The fragments encoding the N-terminal domain of the N protein (N—NTD) and C-terminal domain of the N protein (N—CTD) were then cloned into a modified pET-15b vector in frame with an N-terminal 6 × His-tag and PreScission Protease (PPase) site. The 3CL^pro^ gene was integrated into a modified pGEX-6p-1 vector containing an N-terminal GST-tag, a self-cleavage site AVLQ↓SG [24], and a C-terminal PPase site followed by a 6 × His-tag. Mutants of N—NTD, N—CTD, and 3CL^pro^ were constructed using the QuikChange site-directed mutagenesis kit. The abovementioned plasmids were utilized as templates in this process, as described previously [25].

### 2.2 Protein expression and purification

Plasmids encoding the proteins of interest were transformed into *E. coli* BL21 (DE3) and the transformed cells were cultured at 37 °C in Luria broth, containing a final concentration of 100 μg/ml ampicillin. To induce protein expression, 0.2 mM of isopropyl β-d-1-thiogalactopyranoside was added to the bacterial cultures and incubated at 16 °C for 20 h. Subsequently, cells were harvested and pellets were resuspended in lysis buffer containing 25 mM Tris–HCl at pH 8.0 and 300 mM NaCl. The cells were homogenized with a high-pressure cell disrupter at 4 °C. Lysates were centrifuged at 25,200 × *g* for 50 min at 4 °C, and supernatants were loaded onto a Ni-NTA affinity chromatography column for purification. The proteins were subjected to on-column tag cleavage using PPase (purified in our laboratory) and eluted with lysis buffer. Protein purity was determined using sodium dodecyl sulfate-polyacrylamide gel electrophoresis (SDS-PAGE).

### 2.3 Protein thermal stability assessment

To explore the thermal stability of the proteins, a Protein Thermal Shift™ study was conducted using the Protein Thermal Shift™ Dye Kit from Thermo Fisher Scientific, Shanghai, China. The experiment was performed on an Applied Biosystems QuantStudio 3 (Thermo Fisher Scientific).

In this study, 20 μL of protein sample, with a concentration of 0.5 µg/µL, was combined with 1 × Dye, 20 mM Tris–HCl at pH 8.0, and 100 mM NaCl. The resulting mixture was subjected to gradual heating from 15 °C to 99 °C, at a precise rate of 0.15 °C/s.

To determine the melting temperature (Tm), ROX fluorescence melting curves and the Boltzmann method were employed. The data obtained from triplicate experiments were then utilized to calculate the Tm values, utilizing Protein Thermal Shift™ Software 1.4. Fitting the data in the analysis region to the Boltzmann equation facilitated this crucial determination.

### 2.4 Enzymatic kinetic studies with SARS-CoV-2 3CL^pro^

Kinetic studies of SARS-CoV-2 3CL^pro^ enzymatic activity were investigated using a fluorescence resonance energy transfer (FRET) assay. The FRET substrate MCA-AVLQSGFR-lys (Dnp)-Lys-NH2 was acquired from Beyotime Biotechnology, Shanghai, China. This substrate holds the nsp4/nsp5 cleavage sequence, AVLQ↓SG, making it a versatile peptide substrate suitable for various coronavirus proteases, including the SARS-CoV-2 3CL^pro^.

The proteolytic assay was performed in the buffer containing 20 mM Tris–HCl (pH 7.5), 100 mM NaCl, and 1 mM EDTA. The final concentrations of 3CLpro and FRET-substrate in the 10 μL assay volume were set at 400 nM and 15 μM, respectively. Reaction mixtures were transferred to a black 384-well analytical microplate (Corning, NY, USA). Subsequently, the fluorescence signal, monitored at 25 °C with excitation/emission wavelengths of 340/460 nm, was employed to track the generation of cleavage products.

To evaluate enzyme activity, each mutant protease was incubated at varying temperatures (4 °C, 33 °C, 37 °C, and 39 °C) for 60 min prior to detection of residual enzymatic activity. Moreover, to establish a baseline, wild-type 3CL^pro^ was incubated at 4 °C to be assumed to be its 100% catalytic activity (*k_cat_*). This facilitated the calculation of the relative *k_cat_* of the proteins incubated at different temperatures for various mutations. Each experiment was conducted in triplicate to ensure robustness and accuracy.

## 3 Results

### 3.1 Identification of candidate amino acids for mutagenesis based on protein structural analyses

**Fig. 1:**
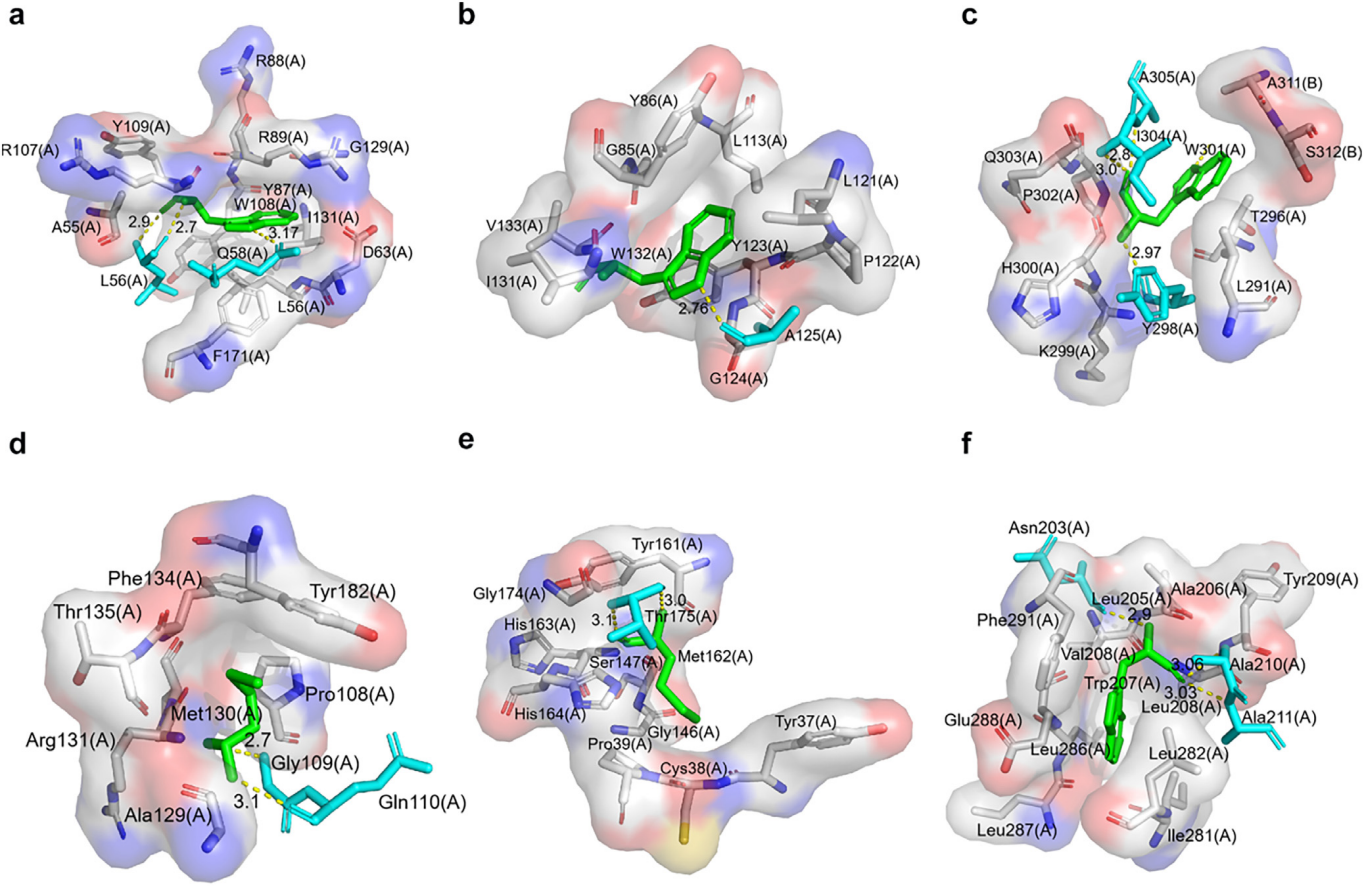
Structure-based analyses of interactions with selected amino acids. (**a**) W108 in N—NTD, (**b**) W132 in N—NTD, (**c**) W301 in N—CTD, (**d**) M130 in 3CL^pro^, (**e**) W162 in 3CL^pro^, and (**f**) W207 in 3CL^pro^. Green depicts the selected tryptophan or methionine residue, while blue represents amino acids engaged in hydrogen bonding with the analyzed residues. Other displayed amino acids indicate hydrophobic residues.

Structural analysis identified two tryptophan residues, W108 and W132, located in the core of the N—NTD structure. W108 forms hydrogen bonds with Q58 and L56, and forms hydrophobic interactions with A55, Y87, R88, R89, R107, G129, and F171. Similarly, W132 engages in hydrogen bonding with A125, and is stabilized through hydrophobic interactions with G85, G124, Y123, Y86, L113, L121, and P122 (Fig. 1). The N—CTD contains a single tryptophan residue, W301. As shown in Fig. 1c, W301 interacts via hydrogen bonds with Y298, I304, and A305, while forming hydrophobic interactions with K299, Q303, Y296, and I291 in the same protomer. Additionally, it engages in hydrophobic interactions with the A311 and S312 residues of an interacting protomer.

The main proteinase (3CL^pro^), contains three tryptophan residues: W31, W207, and W218. W218 is strategically located at the hydrophilic interface, W31 lies in close proximity to the active center Cys145-His41, and W207 is embedded within the structure's core. W207 forms hydrogen bonds with N203, A210, and A211, while engaging in hydrophobic interactions with V204, L205, A206, L208, Y209, I281, L282, L286, L287, E288, and F291 (Fig. 1f). Furthermore, the presence of methionine residues in the structural core of 3CL^pro^ also warrants examination. M130 participates in hydrogen bonding with G109 and Q110, and hydrophobic interactions with P108, A129, R131, F134, T135, and Y182 (Fig. 1d–e). M162 forms hydrogen bonds with T175 and engages in hydrophobic interactions with Y37, C38, P39, G146, S147, Y161, H163, H164, and G174.

After the analysis of amino acid positions, W108 and W132 in N—NTD; W301 in N—CTD; and W207, M130, and M162 in 3CL^pro^ were selected for amino acid substitution because they are all situated in their protein's core.

### 3.2 Replacing tryptophan or methionine with other amino acids reduces protein stability

To minimize the probability of reversion mutations, both tryptophan and methionine were mutated into residues encoded by completely different codons. Since tryptophan (Trp, W) is encoded by TGG, we replaced it with leucine (encoded by CTT), proline (by CCT), histidine (by CAT), glutamine (by CAA), isoleucine (by ATT), threonine (by ACT), asparagine (encoded by AAT), lysine (by AAA), valine (by GTT), alanine (by GCT), aspartic acid (by GAT), and glutamic acid (by GAA) respectively. Since methionine is a hydrophobic nonbranched amino acid, we replaced it with alanine.

Wild-type proteins and all mutant proteins were expressed and purified, and SDS-PAGE analyses of purified products are presented in Supplemental Information (S1). Thermal stabilities were evaluated by determining each protein's Boltzmann Tm. Most of these mutants show low solubility, leading to a reduction in the yield of soluble protein. W108P is insoluble. W108K had a slightly higher Tm than wild-type protein. All ten other W108 mutants exhibited a spectrum of Tm reductions, spanning from 10.83 to 20.58 °C. Among the 12 W132 variants, W132D was insoluble, whereas W132K and N—NTD W132E showed the highest Tm values. All nine other W132 mutants exhibited Tm diminutions ranging from 11.9 to 24.67 °C. In the case of 3CL^pro^ mutants, W207P was insoluble, and all 13 other mutants exhibited a notable diminution in their Tm, with reductions spanning from 3.1 to 10.9 °C (Table 1).

**Table 1:** Thermal Stability of Mutant Proteins.

Protein	Tm (°C)	∆Tm (°C)
N-NTD	48.92	0
N—NTD W108L	34.07	−14.85
N-NTD W108P	–	–
N-NTD W108H	38.09	−10.83
N-NTD W108Q	29.13	−19.79
N-NTD W108I	30.9	−18.02
N-NTD W108T	28.64	−20.28
N-NTD W108N	31.58	−17.34
N-NTD W108K	51.62	+2.7
N-NTD W108V	30.42	−18.5
N-NTD W108A	30.72	−18.2
N-NTD W108D	30	−18.92
N-NTD W108E	28.34	−20.58
N-NTD W132L	26.64	−22.28
N-NTD W132P	30.94	−17.98
N-NTD W132H	37.02	−11.9
N-NTD W132Q	31.76	−17.16
N-NTD W132I	24.67	−24.67
N-NTD W132T	32.01	−16.91
N-NTD W132N	32.06	−16.86
N-NTD W132K	56.15	+7.23
N-NTD W132V	26.32	−22.6
N-NTD W132A	33.05	−15.87
N-NTD W132D	–	–
N-NTD W132E	52.98	+3.32
N-CTD	51.78	0
N-CTD W301L	25.3	−26.48
N-CTD W301P	27.79	−23.99
N-CTD W301H	29.12	−22.66
N-CTD W301Q	25.33	−26.45
N-CTD W301I	25.7	−26.08
N-CTD W301T	28.31	−23.47
N-CTD W301N	26.24	−25.54
N-CTD W301K	25.02	−26.76
N-CTD W301V	24.36	−27.42
N-CTD W301A	25.61	−26.17
N-CTD W301D	27.19	−24.59
N-CTD W301E	26.88	−24.9
3CL^pro^	51.3	0
3CL^pro^ W207L	47	−4.3
3CL^pro^ W207P	–	–
3CL^pro^ W207H	47.5	−3.8
3CL^pro^ W207Q	46	−5.3
3CL^pro^ W207I	44.5	−6.8
3CL^pro^ W207T	45.2	−6.1
3CL^pro^ W207N	48.2	−3.1
3CL^pro^ W207K	44.5	−6.8
3CL^pro^ W207V	47.5	−3.8
3CL^pro^ W207A	47.6	−3.7
3CL^pro^ W207D	40.4	−10.9
3CL^pro^ W207E	41.2	−10.1
3CL^pro^ M130A	42	−9.3
3CL^pro^ M162A	41.5	−9.8

In the semantic framework provided, the symbol " - " conveys the observation of protein insolubility, while the notation " -# " signifies a reduction in the Tm by # °C. Conversely, the notation " +# " indicates an elevation in Tm by # °C.

### 3.3 Correlation between enzyme activity of 3CL^pro^ mutant and TM value

Since 3CL^pro^ is a protease, we assessed the impact of mutations on the enzymatic activity of each mutant at different temperatures. Protein samples were incubated at different temperatures for one hour. Remaining catalytic activity was measured at 25 °C. The enzymatic activity of the samples incubated at 4 °C served as a control for comparison. We assayed three temperatures of particular interest: 33 °C represented the upper respiratory tract temperature, 37 °C the lower respiratory tract temperature, and 39 °C represented fever temperature.

**Fig. 2:**
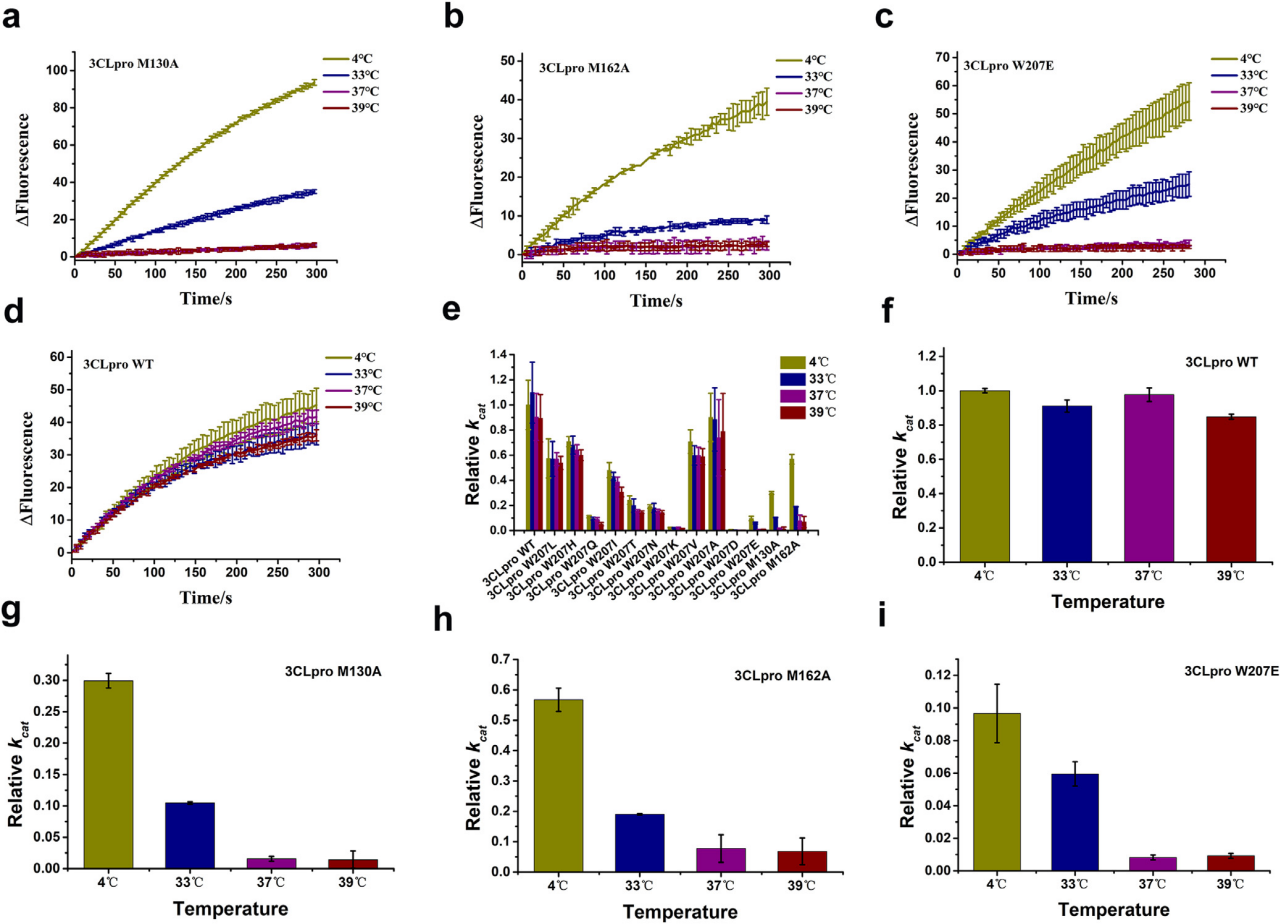
3CL^pro^ enzymatic activity and temperature sensitivity. The different colors used in the curves represent distinct temperatures: green (4 °C), blue (33 °C), purple (37 °C), and red (39 °C). **a–d.** 3CL^pro^ WT (**a**), 3CL^pro^ M130A (**b**), 3CL^pro^ M162A (**c**), 3CL^pro^ W207E (**d**) fluorescence changed with time after incubation at different temperatures for one hour. (**e)** Relative *k_cat_* values of all soluble mutants of 3CL^pro^ at different temperatures. **f–i.** Relative *k_cat_* of 3CL^pro^ WT. (**f**) 3CL^pro^ M130A, (**g**) 3CL^pro^ M162A, (**h**) 3CL^pro^ W207E, and (**i**) incubated at different temperatures of 33 °C, 37 °C, and 39 °C.

The enzyme turnover number (*k_cat_*) reflects the turnover of the substrate at a certain concentration of enzyme per unit time. The *k_cat_* value of wild-type 3CL^pro^ incubated at 4 °C was considered as the reference (set to 1). Based on this value, we calculated the relative *k_cat_* values of the mutants at different temperatures. The results showed reduced activity for most mutants compared to wild-type 3CL^pro^ (Fig. 2e). Wild-type 3CL^pro^ incubated at 33 °C, 37 °C, and 39 °C exhibited basically the same activity as incubated at 4 °C (Fig. 2d, f). Similarly, mutations W207L, W207H, W207Q, W207I, W207T, W207N, W207K, W207V, W207A, M165A, and M276A were not highly sensitive to high temperatures (Fig. 2e). Notably, the catalytic activities of W207E, M130A, and M162A decreased significantly with increasing temperature (Fig. 2a–c). Samples incubated at 37 °C showed activity as low as 10% of that of wild-type 3CL^pro^ (Fig. 2g–i).

## 4 Discussion

The COVID-19 pandemic has profoundly impacted the lives and economic activities of people worldwide. Vaccination has emerged as the most cost-effective and efficient method of protecting vulnerable populations and controlling the spread of infectious diseases. Currently, multiple COVID-19 vaccine formulations have been developed [26], including live attenuated, inactivated, recombinant subunits, vectors, and nucleic acid vaccines. While these vaccines have played crucial roles in combating the pandemic, they all have limitations and their efficacy has fallen short of expectations [[27], [28], [29], [30], [31]]. Therefore, the urgent need to develop more effective and safe vaccines remains.

In this study, we propose a method to reduce the temperature required for viral protein denaturation and envisage its application in temperature-sensitive attenuated vaccines. By analyzing the protein structures of key SARS-CoV-2 components, such as the N protein and 3CL^pro^, we identified specific tryptophans and methionines within each protein's core. By introducing amino acids encoded by completely different codons at this position, temperature-sensitive protein variants were obtained with minimal capability for reversion. Amino acids with a smaller side chain cannot occupy the cavity within a protein structure left by a larger one, leading to decreased Tms compared to their wild-type counterparts.

We used 3CL^pro^ as an example to investigate the relationship between protein denaturation temperature and enzymatic activity. Our results indicate a correlation between Tm and protein activity. When the Tm of the mutant proteins was between 41 °C and 43 °C, the mutant showed approximately 50% activity after incubation at 33 °C but experienced a sharp decrease to only 10% after incubation at 37 °C. Mutant proteins with Tm values higher than 43 °C exhibited minimal sensitivity to high temperatures. In contrast, mutant proteins with Tm values below 41 °C lost most activity even when incubated at 4 °C.

These results validate the feasibility of our approach of engineering mutant strains containing single point mutations or combinations of them with reduced protein denaturation temperatures. We can create strains that can only survive in the nasal cavity or upper respiratory tract but not inside the body, achieving the goal of constructing live attenuated vaccines. In addition, the method used in this study could be applied to the development of temperature-sensitive attenuated vaccines in response to other viruses.

Compared to traditional methods of developing live attenuated vaccines, our approach offers several advantages. It eliminates the need for serial passaging, reduces development time, and allows the administration of thermo-attenuated vaccines through nasal inhalation without the requirement for injections. This strategy thus holds significant promise in the field of live thermo-attenuated vaccine preparation. It is important to note that this study did not involve the construction of variant viral strains or further research on candidate vaccines. Future research should focus on bridging the gap between our research and practical applications.

## 5 Conclusions

We successfully developed a method to reduce the denaturation temperature of key SARS-CoV-2 proteins to a certain point by modifying large amino acids in the cores of folded proteins. A correlation between denaturation temperature and enzyme activity was confirmed. This method can be applied to prepare temperature-sensitive attenuated vaccines to save time and reduce the probability of reversion mutations. These findings provide a foundation for further research on the immunogenicity, efficacy, and safety of thermo-attenuated vaccines against SARS-CoV-2 and other viruses.

## Data Availability Statement

All data generated or analyzed during this study are included in this published article.

## CRediT authorship contribution statement

**Maofeng Wang:** Writing – original draft, Visualization, Methodology, Investigation, Data curation. **Cancan Wu:** Writing – review & editing, Data curation. **Nan Liu:** Writing – review & editing. **Xiaoqiong Jiang:** Data curation. **Hongjie Dong:** Formal analysis. **Shubao Zhao:** Data curation. **Chaonan Li:** Investigation, Data curation. **Sujuan Xu:** Writing – review & editing. **Lichuan Gu:** Writing – review & editing, Supervision, Methodology, Funding acquisition, Conceptualization.

## Declaration of Competing Interest

The authors declare that they have no conflicts of interest with the contents of this article.

## Appendix Supplementary materials

Image, image 1

Image, application 2
