# Erratum to “Regulation of protein thermal stability and its potential application in the development of thermo-attenuated vaccines” [Engineering Microbiology 4 (2024) 100162]

**DOI:** 10.1016/j.engmic.2025.100204

**Published:** 2025-03-25

**Authors:** Maofeng Wang, Cancan Wu, Nan Liu, Xiaoqiong Jiang, Hongjie Dong, Shubao Zhao, Chaonan Li, Sujuan Xu, Lichuan Gu

**Affiliations:** a State Key Laboratory of Microbial Technology, Shandong University, Qingdao 266237, China; b Research Center of Translational Medicine, Central Hospital Affiliated to Shandong First Medical University, Jinan 250013, China; c Department of Orthopedics, Shandong Provincial Hospital Affiliated to Shandong First Medical University, Jinan 250021, China; d Shandong Institute of Parasitic Diseases, Shandong First Medical University & Shandong Academy of Medical Sciences, Jining 272033, China

In the originally published version of this article, the designation of one of the corresponding authors was inadvertently omitted. The article correctly listed **Dr. Lichuan Gu** as a corresponding author, but failed to designate **Dr. Sujuan Xu** as an additional corresponding author. The correct corresponding authors should be:

1. **Dr. Sujuan Xu**
  Email: xu_sujuan@sdu.edu.cn
2. **Dr. Lichuan Gu**
  Email: lcgu@sdu.edu.cn

The publisher regrets this error.

The publisher would like to apologise for any inconvenience caused.

