# Supplementary figures and images for: Regulation of protein thermal stability and its potential application in the development of thermo-attenuated vaccines
Source: Eng Microbiol. 2024 Jun 25;4(3):100162. doi: 10.1016/j.engmic.2024.100162 (PMC11610959; doi:10.1016/j.engmic.2024.100162)

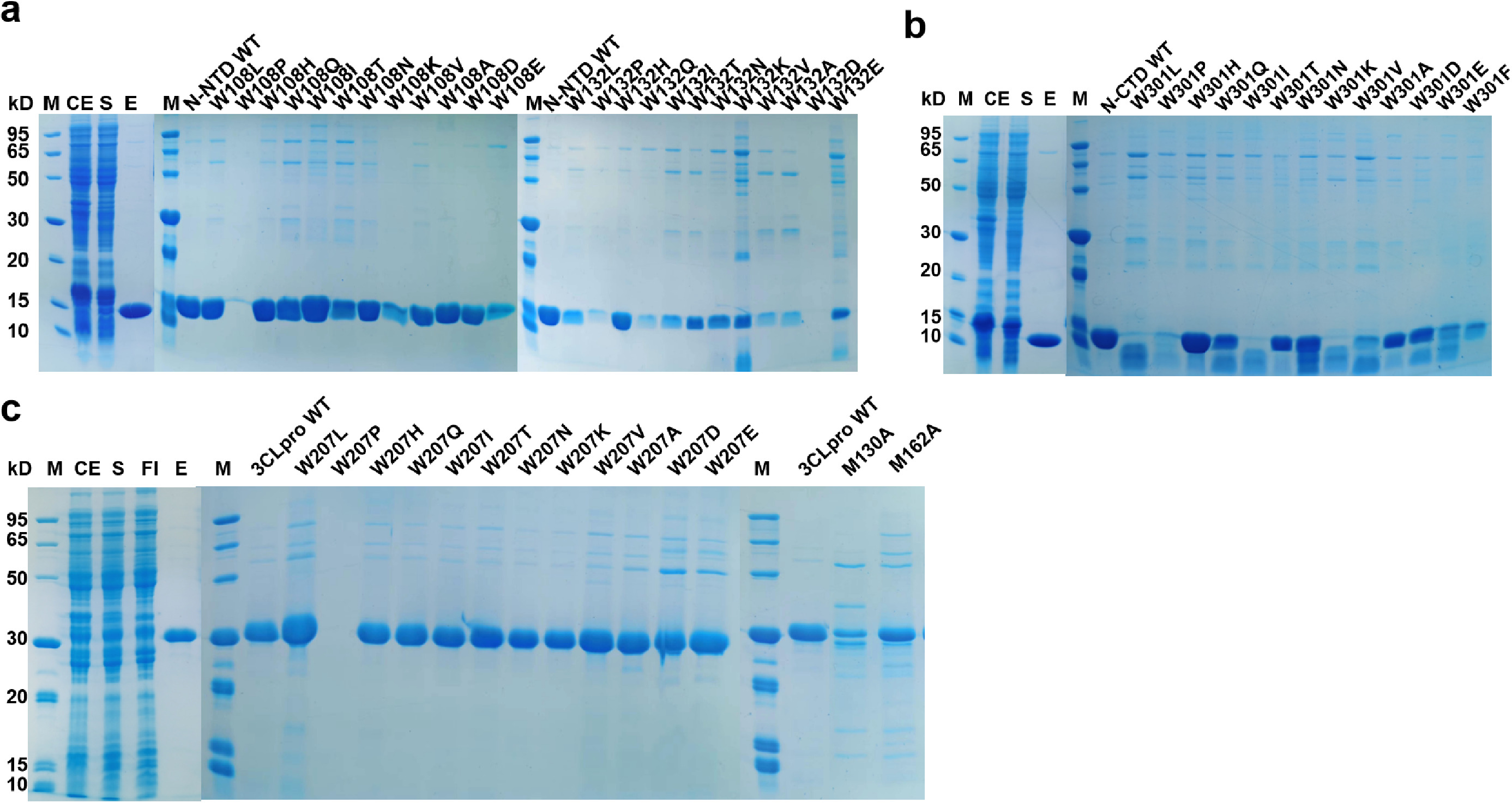

Supplement: Supplementary file 1 [file mmc1.jpg]
