# Supplementary material for: Regulation of protein thermal stability and its potential application in the development of thermo-attenuated vaccines
Source: Eng Microbiol. 2024 Jun 25;4(3):100162. doi: 10.1016/j.engmic.2024.100162 (PMC11610959; doi:10.1016/j.engmic.2024.100162)
Supplement: Supplementary file 2 [file mmc2.doc]

**Regulation of protein thermal stability and its potential application in the development of thermo-attenuated vaccines**

Maofeng Wang^#1, 2^, Cancan Wu^#1^, Nan Liu^3^, Xiaoqiong Jiang^1^, Hongjie Dong^4^, Shubao Zhao^1^, Chaonan Li^1^, Sujuan Xu*^1^ ＆ Lichuan Gu*^1^


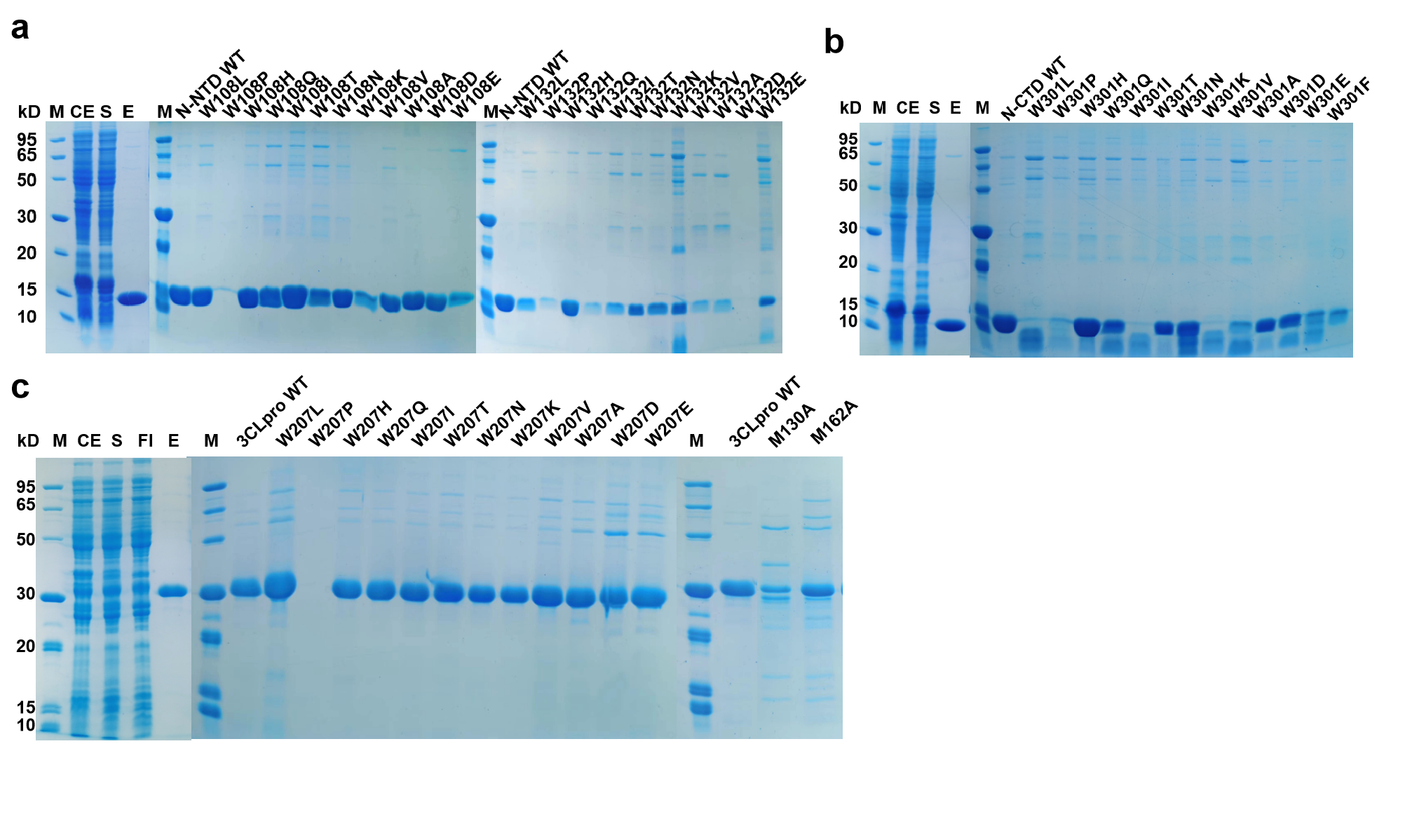


**S1.** The SDS-PAGE results of wild type and mutant proteins. Cell extractive (CE), supernatant (S), flow through (Fl), elution after digestion by PPase (E), and elution of various mutant proteins of N-NTD (**a**), N-CTD (**b**) and 3CLpro (**c**) by Ni affinity chromatography are shown in the figure.
